# Methylation and expression dynamics in broiler liver following *in-ovo* sodium butyrate administration

**DOI:** 10.2478/jvetres-2026-0013

**Published:** 2026-03-13

**Authors:** Aleksandra Beldowska, Elżbieta Pietrzak, Aleksandra Dunisławska

**Affiliations:** Department of Animal Biotechnology and Genetics, Bydgoszcz University of Science and Technology, 85-084 Bydgoszcz, Poland

**Keywords:** embryo stimulation, molecular changes, postbiotic, poultry

## Abstract

**Introduction:**

Butyrate is one of the three main short-chain fatty acids, and it provides energy, controls the state of the intestinal microbiota and mediates the immune response. Sodium butyrate supplementation improves poultry production and changes the intestinal microbiota dynamically. These changes may affect the liver directly and indirectly through pathways in the gut–liver axis, the bidirectional relationship between the liver and intestines. The study analysed gene expression and methylation in the broiler liver after *in-ovo* stimulation by sodium butyrate.

**Material and Methods:**

Incubated Ross 308 broiler eggs were injected on day 12 with saline as the control group or with sodium butyrate as SB groups at three doses: 0.1%, 0.3% and 0.5%. Chicks’ livers were collected postmortem on day 42 of rearing for RNA and DNA extraction. Gene expression was analysed by reverse-transcription qPCR, and gene methylation by methylation-specific qPCR for a panel of lipid metabolism and immune regulation genes comprising *ANGPTL4, CD72, CXCR5, CYR61, IKZF1, KLHL6, NR4A3, SERPING, SYK* and *TNFRSF14*.

**Results:**

Sodium butyrate stimulation changed gene expression levels. Upregulation was noted of *ANGPTL4* and *NR4A3* in the 0.1% and 0.3% SB groups and downregulation of *CYR61* in the 0.5% SB group. The gene-specific DNA methylation also significantly changed under the influence of the sodium butyrate doses for *ANGPTL4, CXCR5* and *KLHL6*. The level of global methylation did not change significantly, but did decrease with increasing butyrate dose.

**Conclusion:**

The obtained results suggest that sodium butyrate affected both gene expression and methylation in the liver, indicating its potential epigenetic effects.

## Introduction

*In-ovo* stimulation has the potential to modulate the expression of genes related to metabolism and development, and consequently, may influence poultry performance. Stimulation of eggs with appropriate nutrients or bioactive substances is a novel way to improve embryo health and accelerate its proper development (34). *In-ovo* technology has been used to deliver various bioactive substances, including carbohydrates, amino acids, hormones, prebiotics, probiotics, synbiotics, antibodies, immunostimulants and minerals to modify the gut microbiome (29).

Manipulation of this microbiome can have beneficial effects on animal welfare. The intestinal mucosa plays a vital role in protecting the host’s internal tissues from pathogens and mechanical damage during digestion, serving as the first line of defence (10). In poultry, maintaining intestinal health is crucial for growth, performance and disease resistance, and dietary supplements offer a non-antibiotic strategy to achieve this. One such supplement is sodium butyrate (SB), one of the short-chain fatty acids (SCFAs) known for lowering intestinal pH, inhibiting harmful microbial colonisation and promoting intestinal villi development (15), while being generally considered a safe antibiotic alternative. The broiler gut microbiota significantly influences nutrient absorption, immune system maturation and overall health (2, 20). Sodium butyrate enhances intestinal integrity by modulating mucosal structure, regulating gene expression and increasing SCFA production. It supports mucin and antimicrobial peptide synthesis, reducing intestinal permeability and promoting beneficial bacteria such as *Lactobacillus* spp. and *Bifidobacterium* spp. (3). Beyond intestinal health, SB impacts liver metabolism, which is central to lipid regulation in poultry. The liver governs lipid absorption, synthesis, β-oxidation and lipoprotein transport, with imbalances potentially leading to fatty liver syndrome (FLS), a common issue in commercial broiler farming associated with increased mortality (35). Nutritional interventions, including choline, betaine and SCFAs, are commonly used to mitigate FLS risk (9).

Butyric acid, the active form of SB, is a microbial fermentation product of undigested food and dietary fibre and serves as an energy source for intestinal epithelial cells (22). It demonstrates anti-inflammatory and antioxidant properties, alleviates intestinal inflammation, improves morphology, and fosters microbial balance (21, 31). Supplementation with coated SB has been shown to reduce hepatic and abdominal fat deposition in broilers and suppress preadipocyte fat accumulation (40), while enhancing antioxidant enzyme activity and mitigating lipid-induced oxidative stress (6). Moreover, SB operates among other ways through epigenetic regulation, leading to lasting phenotypic changes. Epigenetic mechanisms, particularly DNA methylation, play a role in gene expression regulation and are responsive to environmental factors during early development (7, 42). Methylation typically occurs at CpG dinucleotides (cytosine-guanine sites subject to DNA methylation) and, when present in promoter regions, can inhibit gene transcription by blocking transcription-factor binding (38). The genes examined in our research play key roles in lipid metabolism and immune regulation in poultry, particularly in response to exogenous sodium butyrate exposure, including *via in-ovo* supplementation. The angiopoietin-like 4 gene, *ANGPTL4*, regulates plasma triglyceride levels by inhibiting lipoprotein lipase and is responsive to nutritional status in broilers (26). The cluster of differentiation 72 gene, *CD72*, and the spleen-associated tyrosine kinase gene, *SYK*, are involved in B cell receptor signalling, influencing immune activation (8). The signals of the toll-like receptor 4 gene, *TLR4*, in B lymphocytes are transduced *via* the B cell antigen, and the C-X-C motif chemokine receptor type 5 gene, *CXCR5*, guides lymphocyte migration to germinal centres, which is important for adaptive immunity (32). The Ikaros family zinc finger 1 and kelch-like family member 6 genes, *IKZF1* and *KLHL6*, contribute to lymphocyte differentiation and B cell maturation (18). The cysteine-rich angiogenic inducer 61 gene, *CYR61*, is involved in liver regeneration and angiogenesis (41). The nuclear receptor subfamily 4 group A member 3 gene, *NR4A3*, regulates energy metabolism and inflammatory responses in avian species, and the serpin family G member 1 and tumour necrosis factor receptor superfamily 14 genes, *SERPING1* and *TNFRSF14*, are key regulators of innate and adaptive immunity (11, 23). The present study aimed to investigate the expression of these genes and the methylation of DNA in the liver of broiler chickens following *in-ovo* stimulation with sodium butyrate.

## Material and Methods

### Experimental design

One thousand hatching eggs of Ross 308 broiler chickens were obtained from the Radomice Poultry Hatching Plant (Radomice, Poland). On day 12 of incubation, the eggs were randomly allocated into four groups of 250 eggs each. These groups were injected into the air chamber with 0.2 mL of either physiological saline (control) or a solution of sodium butyrate in physiological saline at a concentration of one of 0.1%, 0.3% or 0.5% (110.09 g/mol; Merck Life Science, Poznań, Poland). The eggs were incubated for 21 d. Upon hatching, 60 chicks of similar body weight were selected from each group for rearing. Each group was kept in five replicates of 12 chicks each. On the first day of rearing, the temperature was 30°C, and it was lowered gradually to come down to 20°C in the last week. An additional heat source providing a temperature 2°C higher than the ambient building temperature was used for the first four weeks. The humidity was approximately 60%. The rearing facility was prepared 24 h before, and chopped wheat straw was used as bedding. The lighting schedule consisted of 18 h of light and 6 h of darkness, with extended light periods of 23 h during the first and last three days of rearing. The chicks had free access to fresh water and feed, accessible in each pen from a bellshaped drinker and a wall-mounted feeder. The rearing and production data were published in the description of a companion study by Bełdowska *et al*. (4).

The feed was purchased from a commercial supplier and was balanced to the nutrition standards for broiler chickens. Three feeding phases were used: starter feed from day 1 to day 14, grower feed from day 15 to day 35 and finisher feed from day 36 to day 42 of rearing. The commercial diets provided to the broilers contained all essential nutrients, including a complete vitamin and mineral premix. The starter diet was supplied in crumble form and contained (per kg): 19.70% crude protein, 4.00% crude fat, 4.20% crude fibre, 5.30% crude ash, 1.14% lysine, 0.52% methionine, 0.80% calcium, 0.51% phosphorus and 0.15% sodium. Its formulation included corn, wheat, soybean meal, wheat bran, triticale, dehulled sunflower meal, rapeseed meal, calcium carbonate, animal fat, monocalcium phosphate, sodium chloride and sodium bicarbonate. The grower diet, also in crumble form, contained 18.40% crude protein, 4.00% crude fat, 3.50% crude fibre, 4.40% crude ash, 1.01% lysine, 0.46% methionine, 0.60% calcium, 0.38% phosphorus and 0.15% sodium, and was composed of corn, wheat, soybean meal, triticale, dehulled sunflower meal, rapeseed meal, calcium carbonate, animal fat, sodium chloride and sodium bicarbonate. The finisher diet, provided in pelleted form, contained 17.30% crude protein, 3.50% crude fat, 3.90% crude fibre, 4.30% crude ash, 0.95% lysine, 0.42% methionine, 0.60% calcium, 0.40% phosphorus and 0.16% sodium, and included corn, triticale, soybean meal, dehulled sunflower meal, rapeseed meal, calcium carbonate, animal fat, sodium chloride and sodium bicarbonate. An average metabolisable energy content was 12.50 MJ/kg.

### Sample collection

After reaching day 42, eight broiler chickens were randomly selected from each control and experimental group. After sacrifice, liver samples were taken for RNA and DNA isolation and were immediately placed in a stabilisation buffer (fixRNA; EURx, Gdańsk, Poland). After transport, all samples were stored at –80°C in a laboratory freezer and stored for analyses.

### Relative gene expression in the liver

Total RNA was isolated from approximately 150 mg of liver tissue, which had been homogenised in 1 mL RNA Extracol (EURx). For each mL of extract, 0.2 mL of chloroform was added, and then the tissue was homogenised using a TissueRuptor homogeniser (Qiagen, Hilden, Germany). Following the manufacturer's protocol, RNA was purified using the GeneMATRIX Universal RNA Purification Kit (EURx). Each RNA sample was assessed for quantity and quality using a NanoDrop 2000 spectrophotometer (Thermo Fisher Scientific, Wilmington, DE, USA). Gene expression analysis was conducted *via* qPCR, starting with reverse transcription of RNA for each sample. Compliment DNA was synthesised using the smART First Strand cDNA Synthesis Kit (EURx). The qPCR reaction mixture contained SG (SYBR Green) onTaq qPCR Master Mix (2×) (EURx), 1 μM of each primer (synthesised by Merck, Darmstadt, Germany) and 70 ng of cDNA. Reactions were performed in a LightCycler 480 II thermal cycler (Roche Diagnostics, Basel, Switzerland) with the following thermal profile: initial denaturation at 95°C for 15 min followed by 40 cycles of 95°C for 15 s, 58°C for 15 s and 72°C for 45 s. A melting curve analysis was conducted at the end of each run. Relative gene expression was calculated using the formula 2^–△△CT^ (27). Based on microarray data published by Dunisławska *et al*. (13), reference genes and an experimental gene panel were selected for expression analysis (Table 1).

**Table 1.** Primers for the panel of genes used to measure relative gene expression

Gene	Name	Forward/reverse primer sequences ()	Reference
ACTB	actin β	F: CACAGATCATGTTTGAGACCTT R: CATCACAATACCAGTGGTACG	12
GAPDH	glyceraldehyde 3-phosphate dehydrogenase	F: GGCACGCCATCACTATC R: CCTGCATCTGCCCATTT	12
ANGPTL4	angiopoietin-like 4	F: TCCTCGATTCGCGAGTTCTG R: CAGGGCACTGGGAGCTG	12
CD72	cluster of differentiation 72	F: AGGAAGGTAGGGCAGCAATG R: CTGACCTGAGGTTCGCCAAA	14
CXCR5	C-X-C motif chemokine receptor 5	F: GCTCTGACTGTAGGGTGACG R: TGAAATGATGGGCAGTGGCT	14
CYR61	cysteine-rich angiogenic inducer 61	F: ATCGCTCGTTCAGACGCATA R: TGTCTGGGCTCCGCTAAAAG	12
IKZF1	Ikaros family zinc finger 1	F: GCGTGTGAAAGAGCGACTTC R: GAACACTCCGCACAACACCT	14
KLHL6	kelch-like family member 6	F: ATGGTTTCTGCGTCAACTCC R: CATCCTGGCTGGGATGCAATA	12
NR4A3	nuclear receptor subfamily 4 group A member 3	F: GGCATCCCCGGAGTTTCTCTG R: TTTGACGAGGCCGCTCATT	12
SERPING1	serpin family G member 1	F: GTCCTCGTGCCACACTTACC R: TTGACCAATGCTTGCCCACC	14
SYK	spleen-associated tyrosine kinase	F: AAGGGACAGCAATGGTTCCT R: AATTTAACAGACCTGCCAGAGG	12
TNFRSF14	tumour necrosis factor receptor superfamily member 14	F: TGAGCACCATCAGGGGTATC R: AGGTACGGATGCTTCCCAAG	14

### DNA methylation in the liver

Liver DNA was isolated following the manufacturer’s protocol using the GeneMATRIX Tissues DNA Purification Kit (EURx). The quality and quantity of the isolated DNA were assessed using the NanoDrop 2000 spectrophotometer. The DNA was then bisulphite-converted using the CiTi Converter DNA Methylation Kit (A&A Biotechnology, Gdańsk, Poland) according to the manufacturer’s instructions. The qPCR reaction for selected genes was conducted using a LightCycler 480 thermal cycler. The reaction mixture comprised the dye from the SG onTaq qPCR Master Mix kit (EURx), 1 μM of each primer (synthesised by Merck, Darmstadt, Germany) and 50 ng of converted DNA. The optimised melting point was 58°C. After amplification, a melting curve was generated for each product. The relative DNA methylation level (%) was calculated from the melting curve fluorescence level readings for each sample using the following formula, according to Fackler *et al*. (16):

$$\%\text{ of methylation }=100\times \left(\frac{M}{M+U}\right)$$

where M – average fluorescence intensity of the methylated product and U – average fluorescence intensity of the unmethylated product.

The panel of genes used in the analysis is presented in Table 2. Global methylation was calculated according to the instructions from the MethylFlash Methylated 5mC DNA Quantification Kit (Colorimetric) (EpigenTek, Farmingdale, NY, USA).

**Table 2.** Primers for the panel of genes used for DNA methylation analysis

Gene	Name	Methylated (M)/unmethylated (U) Forward (F)/Reverse (R) primer sequences	Reference
ANGPTL4	angiopoietin-like 4	MF: TAATTTTAACGGGAAGTATTTTCGT MR: CAACTTTAAAACTCTACCTCCAACG UF: TAATTTTAATGGGAAGTATTTTTGT UR: ACTTTAAAACTCTACCTCCAACACA	12
CD72	cluster of differentiation 72	MF: AACGGGTTATGTGTCGTTATTAGTC MR: AAACTAAACCCTACTACCTTCTCGC UF: TGGGTTATGTGTTGTTATTAGTTGT UR: ACTAAACCCTACTACCTTCTCACA	12
CXCR5	C-X-C motif chemokine receptor 5	MF: AGAGGTTGGGATTTACGGTAATAAC MR: ACAACTTTCTACCTTTACAAACGCT UF: AGGTTGGGATTTATGGTAATAATGT UR: ACAACTTTCTACCTTTACAAACACT	12
CYR61	cysteine-rich angiogenic inducer 61	MF: TTTGGTTTTAGTGTTTAAAGACGT MR: TTATATTTACCTTCAAAAAAACGTA UF: TTTTGGTTTTAGTGTTTAAAGATGT UR: TATTTATATTTACCTTCAAAAAAACATA	12
IKZF1	Ikaros family zinc finger 1	MF: GTAGTAGTAATTGTTGGAGGAGGC MR: AAAAATAACTTTACGAAACAACGAA UF: GTAGTAGTAATTGTTGGAGGAGGTG OR: AAAAATAACTTTACAAAACAACAAA	12
KLHL6	kelch-like family member 6	MF: TTTTTTGGATAATGAGTGTTTAACG MR: AAACACCAAAAAAAATCCCGTA UF: TTTTTGGATAATGAGTGTTTAATGA UR: CTAAAACACCAAAAAAAATCCCATA	12
NR4A3	nuclear receptor subfamily 4 group A member 3	MF: GGGAAAGGATAAAGTTTTTGTAGTC MR: AAACTCAAACGTAACCCTAAACGTA UF: GGGAAAGGATAAAGTTTTTGTAGTTG UR: AAACTCAAACATAACCCTAAACATA	12
SERPING1	serpin family G member 1	MF: GGTAACGAGAGTTTGGATTTGTAAC MR: CCTAAATAAACCCTAAAAACTACGC UF: TGGTAATGAGAGTTTGGATTTGTAAT UR: CTAAATAAACCCTAAAAACTACACC	12
SYK	spleen-associated tyrosine kinase	MF: TATTAGGCGTTTTCGGGAAC MR: AAATTAATACATTTACTCGCCGCT UF: GTTTATTAGGTGTTTTTGGGAATGA UR: CCAAATTAATACATTTACTCACCACT	12
TNFRSF14	tumour necrosis factor receptor superfamily member 14	MF: GTTTTAGTTATTTTTGTTTTTACGTTCGT MR: CCGCTATCACTATACAACTTCTCG UF: AGTTATTTTTGTTTTTATGTTTGT UR: CACTATCACTATACAACTTCTCACC	12

### Statistical analysis

The differentiating factor in the statistical analysis was the dose of sodium butyrate (SB). Data distribution was assessed within groups using the Shapiro–Wilk test and variance homogeneity was verified with the Brown–Forsythe test. Based on the results, the analysis of relative gene expression levels and global DNA methylation was conducted using a one-way ANOVA, followed by Tukey’s multiple comparison test for *post-hoc* pairwise analysis. Data derived from the targeted DNA methylation analysis did not fully meet the assumptions of normal distribution; therefore, a nonparametric Kruskal–Wallis test was applied, followed by Dunn’s multiple comparison test to identify significant intergroup differences. Data are expressed as mean with SEM, and differences were considered statistically significant at P-value < 0.05. All statistical analyses and graphical visualisations were performed using GraphPad Prism software version 9.0.0 (GraphPad Software, San Diego, CA, USA).

## Results

### Gene expression level

**Fig. 1.**
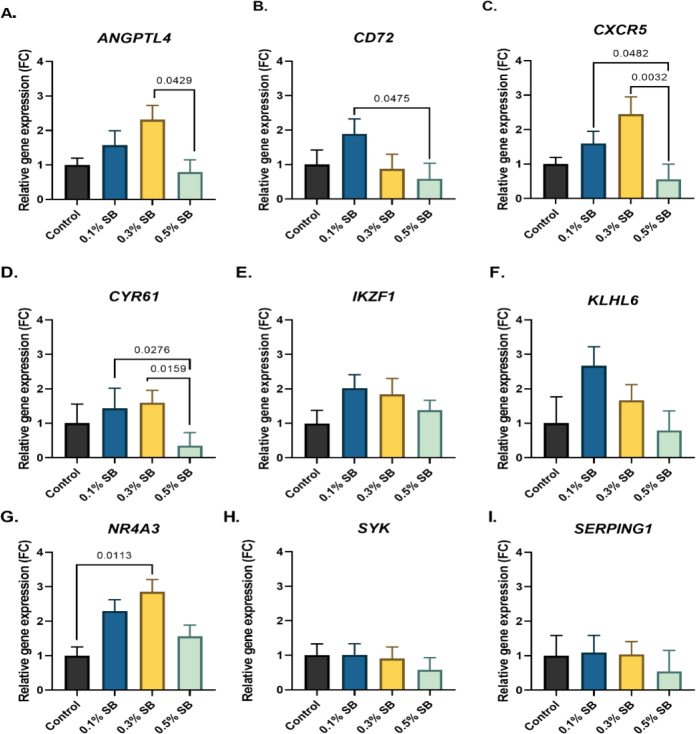
Relative expression of selected genes in the liver of 42-day-old Ross 308 broilers after *in-ovo* sodium butyrate administration on day 12 of incubation. Values above 1 indicate upregulation, and values below 1 indicate downregulation compared to the control. FC – fold change; 0.1% SB – group administered 0.1% sodium butyrate; 0.3% SB – group administered 0.3% sodium butyrate; 0.5% SB – group administered 0.5% sodium butyrate; ANGPTL4 – angiopoietin-like 4; CD72 – cluster of differentiation 72; CXCR5 – C-X-C chemokine receptor type 5; CYR61 – cysteine-rich angiogenic inducer 61; IKZF1 – Ikaros transcription factor; KLHL6 – kelch-like family member 6; NR4A3 – nuclear receptor subfamily 4 group A member 3; SYK – spleen-associated tyrosine kinase; SERPING1 – serpin family G member 1. Data are presented as mean SEM (n = 8). Exact P-values shown above the brackets indicate significant differences between specific treatment groups (P-value < 0.05)

The expression of the *ANGPTL4* gene in the 0.3% group was more than twice as high as in the 0.5% group. The *CD72* gene’s expression differed significantly between the 0.1% group, where it was highest, and the 0.5% group. The *CXCR5* gene showed expression differences between the group with the highest SB concentration and all remaining groups but the control group. In the case of *CYR61*, the expression level was several times lower in the 0.5% group than in the group with the lowest SB concentration, but was similar in the 0.3% group and the 0.5% group. The expression of the *NR4A3* gene showed a significant deficit in the 0.3% group compared to the control. For the *IKZF1* gene, the expression level did not differ significantly between groups. The *KLHL6* gene was expressed the most in the 0.1% group, but between no groups was the expression difference significant. The *SERPING1* and *SYK* gene levels in each group were similar and differed only well below the significance level. No detection was achieved of the *TNFRSF14* gene. All gene expression results are shown in Fig. 1.

### DNA methylation level

**Fig. 2.**
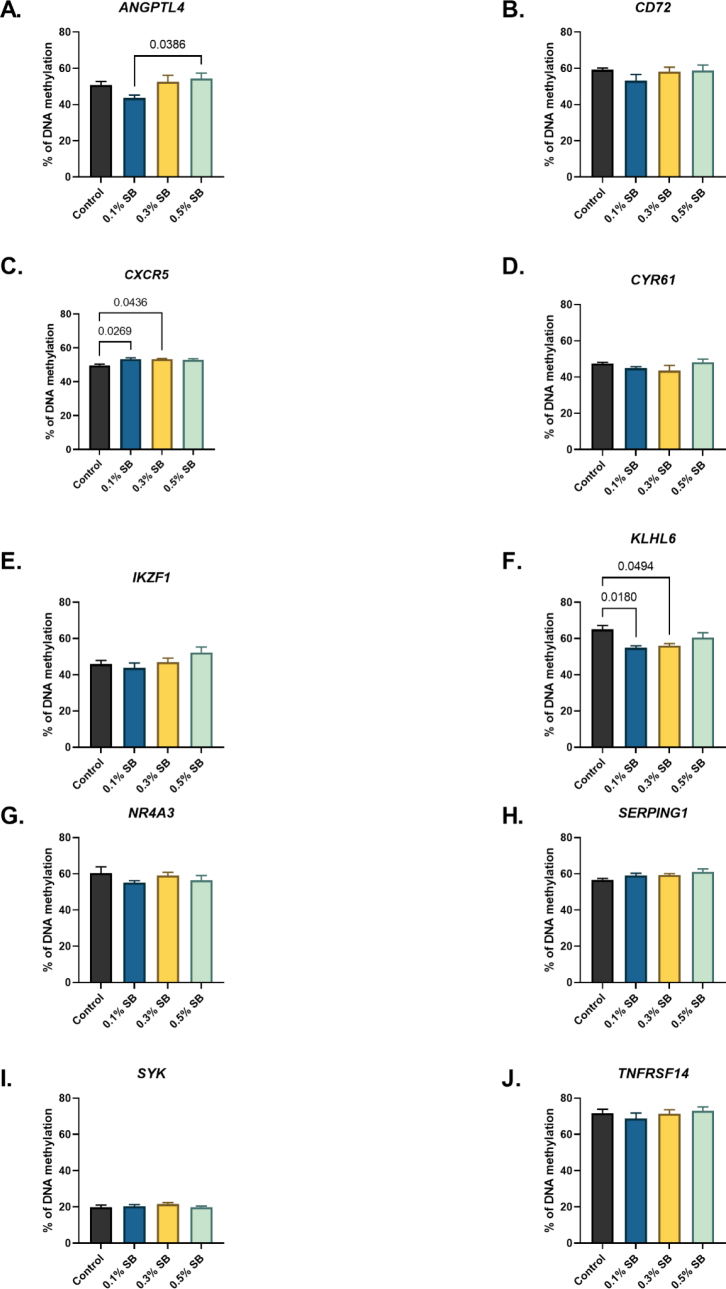
Extent of DNA methylation of selected genes in the liver of 42-day-old Ross 308 broilers after *in-ovo* sodium butyrate administration on day 12 of incubation. 0.1% SB – group administered 0.1% sodium butyrate; 0.3% SB – group administered 0.3% sodium butyrate; 0.5% SB – group administered 0.5% sodium butyrate; ANGPTL4 – angiopoietin-like 4; CD72 – cluster of differentiation; CXCR5 – C-X-C chemokine receptor type 5; CYR61 – cysteine-rich angiogenic inducer 61; IKZF1 – Ikaros transcription factor; KLHL6 – kelch-like family member; NR4A3 – nuclear receptor subfamily 4 group A member 3; SERPING1 – serpin family G member 1; SYK – spleen-associated tyrosine kinase; TNFRSF14 – tumour necrosis factor receptor superfamily member 14. Data are presented as mean and SEM (n = 8). Exact P-values shown above the brackets indicate significant differences between specific treatment groups (P-value < 0.05)

Significant deviations in the level of DNA methylation in the liver occurred only for three studied genes (*ANGPTL4, CXCR5* and *KLHL6*). For the *ANGPTL4* gene, significance was observed in the difference between the methylation in the lowest dose group and methylation in the highest dose group. A low but significant increase in the methylation level compared to the control was also observed for *CXCR5* in the 0.1% and 0.3% groups. The methylation level of the *KLHL6* gene was significantly lower in these groups than in the control group. For the remaining genes, methylation levels did not differ significantly between groups. All methylation results are shown in Fig. 2.

**Fig. 3.**
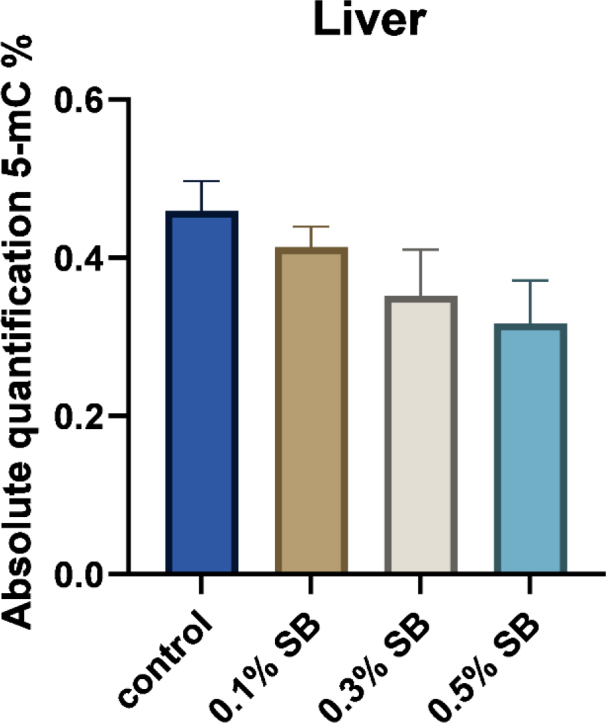
Global DNA methylation levels in the liver of 42-day-old Ross 308 broilers after *in-ovo* sodium butyrate administration on day 12 of incubation. 5-mC – 5-methylcytosine; 0.1% SB – group administered 0.1% sodium butyrate; 0.3% SB – group administered 0.3% sodium butyrate; 0.5% SB – group administered 0.5% sodium butyrate. Data are presented as mean and SEM (n = 8). No significant effect was noted on global DNA methylation (P-value = 0.1441)

## Discussion

The current research builds upon previous work by Bełdowska *et al*. (4), in which it was demonstrated that *in-ovo* stimulation of sodium butyrate on day 12 of egg incubation modulated the gut microbiota and influenced gene expression in the intestinal mucosa of broiler chickens. In that study, changes in caecal microbiota were accompanied by altered expression of genes associated with immune response and barrier function. While microbiome analyses were not included in the current publication, a related but distinct aspect was explored: the long-term epigenetic effects of *in-ovo* stimulation with sodium butyrate on liver gene expression and DNA methylation level. Recognising the functional relationship of the gut–liver axis, we aimed to investigate whether early-life microbiota and intestinal changes may be associated with downstream molecular responses in the liver. Research conducted by Villaluenga *et al*. (37) showed that the 12^th^ day of egg incubation was the optimal injection date for raffinose family oligosaccharides because it produced the highest level of bifidobacteria in chick faeces. This date is also supported by its proximity to the subsequent episode of rapid growth of the chicken embryo, during which the consumption of yolk fat increases dramatically, and the absorption of nutrients increases intensively. At the same time, the yolk sac decreases (29). The 42^nd^ posthatch day is considered a standard endpoint in broiler studies, as it corresponds to the typical market age and the period of maximal physiological growth, during which broilers increase in body weight approximately 50-fold. This time point is optimal for evaluating longterm nutritional and metabolic effects, reflecting peak nutrient demand and commercial relevance in poultry production systems (28, 36). Studies conducted by Akram *et al*. (1) have shown a dependence of the level of gene expression in that case *CLDN1, TJAP1, IL10, IL12p40* and *MUC6* and the same with number of bacteria such like *Lactobacillus* spp., *Ruminococcaceae, Faecalibacterium, Romboutsia* and *Tyzzerella* on the size of the SB dose. They have also been proved that the group with the addition of 0.3% SB showed the highest microbiological diversity 14 d after hatching and the most favourable bacterial profiles at all time points. Bawish *et al*. (3) found that high doses of SB induced levels of expression of *the insulin-like growth factor 1* and *toll-like receptor 4* genes in the liver which could correlate with better growth and activation of the immune system. In our study, higher expression of almost all tested genes was observed in the groups with the addition of 0.1% and 0.3% than in the control group. In comparison, in the 0.5% group, the expression level of most genes was significantly lower than in the control group. This effect may result from an excessive dose of butyrate.

Methylation of DNA affects gene function by activating molecular mechanisms related to biological and disease processes. It contributes to the silencing of gene expression because it changes the structure of chromatin into an inactive and condensed form known as heterochromatin. Many methylated nucleotides act as a signals for chromatin-building proteins, which organise the beginning of chromatin condensation processes (24). The *ANGPTL4* gene is responsible for encoding a protein that regulates glucose homeostasis, insulin sensitivity, lipid metabolism and the regulation of food intake (43). In the group given the lowest dose, the expression level of this gene was upregulated, but methylation was less intense, while in the group given the highest dose, methylation was more intense, and as a result, expression was downregulated. This can be attributed to the phenomenon of prevention of the binding of transcription factors to promoters through the methylation process, which, in effect, hinders the transcription of a given gene and reduces its expression level (44). A similar situation was observed for the *CYR61* gene. In the groups in which the expression was stronger, the methylation was weaker. The protein encoded by *CYR61* has a role in cell proliferation, apoptosis and differentiation. In addition, it is a growth factor promoting endothelial cell adhesion (19). In the studies described by Dunisławska *et al*. (14), downregulation of *IKZF1* and *NR4A3* gene expression was associated with greater gene methylation. In our study, the decrease in *KLHL6* methylation levels relative to controls coincided with an increase in gene expression in the study groups. In the case of this gene, which is involved in B lymphocyte development, this may indicate activation of B cell maturation. In the case of the *TNFRSF14* gene, which showed a methylation level of approximately 80% after *in-ovo* stimulation with sodium butyrate but which was not expressed, this finding is quite surprising and may indicate that specific methylation locations may play key roles in the regulation of gene expression. Nätt *et al*. (33) reported that there may be a significant functional difference between methylation at transcription factor binding sites and methylation of sequences that function as insulators. Although it is often assumed that methylation of promoter regions correlates with transcriptional repression, their results indicate that the control of gene expression can be modulated by a wider range of mechanisms, including chromatin structure. However, further research into the mechanisms of epigenetic regulation is necessary. Importantly, DNA methylation is not functionally uniform across the genome; methylation within promoter-associated CpG islands is typically linked to transcriptional silencing, whereas methylation occurring in gene bodies or distal regulatory elements may have context-dependent or even transcription-permissive effects (39). Our study observed that higher expression of the *NR4A3* gene was associated with less methylation. The chemokine receptor *CXCR5* is a major regulator of B-cell trafficking and T-cell subsets. This chemokine can recruit immune cells to the site of infection (25). The *CXCR5* gene may be involved in immune cell migration. Studies conducted by Hong *et al*. (20) have shown that *CXCR5* expression levels in the bursa of Fabricius decrease following injection of lipopolysaccharides. In our studies, the level of *CXCR5* methylation in the liver exceeded the control level in the groups stimulated with SB, which may be associated with less expression of this gene in the stimulated groups. During embryogenesis, DNA demethylation (the reverse process of methylation, in which a methyl group is removed from the molecule) occurs immediately after zygote formation. A new methylation profile begins *de novo* from the blastocyst stage (30). In the studies of Gryzińska *et al*. (17), it was proved that methylation is related to age. An increase in global DNA methylation in embryos was shown between the 6^th^ and 18^th^ days of embryonic development. Chang *et al*. (5) showed that the DNA methylation and chromatin density ratios in the promoter region of *GPR41/43* genes were changed after sodium butyrate administration. This indicates that sodium butyrate may participate in DNA methylation and chromatin remodelling, regulating target gene expression *via* an epigenetic mechanism. In our studies, with increasing butyrate dose, the level of global methylation decreased.

This study demonstrates that *in-ovo* administration of sodium butyrate had long-term, dose-dependent effects on liver gene expression and DNA methylation in broiler chickens. The lower doses (0.1% and 0.3%) upregulated the expression of key genes involved in metabolism and immune function, while the highest dose (0.5%) downregulated it, likely because of excessive DNA methylation which limited transcription. The observed inverse relationship between methylation and gene expression – particularly for *ANGPTL4, CYR61, NR4A3* and *CXCR5* – confirms that epigenetic mechanisms regulate these processes. The choice of the 12th day of incubation as the injection point is supported by evidence in previous research of optimal microbial colonisation and rapid embryonic growth. The effects observed at day 42 post hatching indicated that early-life intervention with sodium butyrate can induce lasting molecular changes, reflecting the functional connection between the gut and the liver. The observed changes in the expression and methylation of the selected genes appear to be ultimately beneficial, particularly at the lower doses of sodium butyrate (0.1% and 0.3%). This obtained results do not clearly indicate an increase in broiler performance; however, the changes in the level of gene expression and methylation have a positive effect on immunological and metabolic processes, which may contribute to reducing the impact of stress factors and thus to improving the welfare and health of animals.

## Conclusion

The findings suggest that carefully controlled dosing of sodium butyrate may offer a strategy to modulate metabolic and immune pathways in poultry through epigenetic regulation. Epigenetic regulation of gene expression under the influence of postbiotics *in-ovo* requires further study and is an important thread in the context of the gut–liver axis in poultry.
